# Chemical approaches to the sulfation of small molecules: current progress and future directions

**DOI:** 10.1042/EBC20240001

**Published:** 2024-12-04

**Authors:** Jaber A. Alshehri, Alan M. Jones

**Affiliations:** School of Pharmacy, University of Birmingham, Edgbaston, B15 2TT, United Kingdom

**Keywords:** Metabolism, Sulfation, Synthesis

## Abstract

Sulfation is one of the most important modifications that occur to a wide range of bioactive small molecules including polysaccharides, proteins, flavonoids, and steroids. In turn, these sulfated molecules have significant biological and pharmacological roles in diverse processes including cell signalling, modulation of immune and inflammation response, anti-coagulation, anti-atherosclerosis, and anti-adhesive properties. This *Essay* summarises the most encountered chemical sulfation methods of small molecules. Sulfation reactions using sulfur trioxide amine/amide complexes are the most used method for alcohol and phenol groups in carbohydrates, steroids, proteins, and related scaffolds. Despite the effectiveness of these methods, they suffer from issues including multiple-purification steps, toxicity issues (e.g., pyridine contamination), purification challenges, stoichiometric excess of reagents which leads to an increase in reaction cost, and intrinsic stability issues of both the reagent and product. Recent advances including SuFEx, the *in situ* reagent approach, and TBSAB show the widespread appeal of novel sulfating approaches that will enable a larger exploration of the field in the years to come by simplifying the purification and isolation process to access bespoke sulfated small molecules.

## Introduction

**Figure 1:**
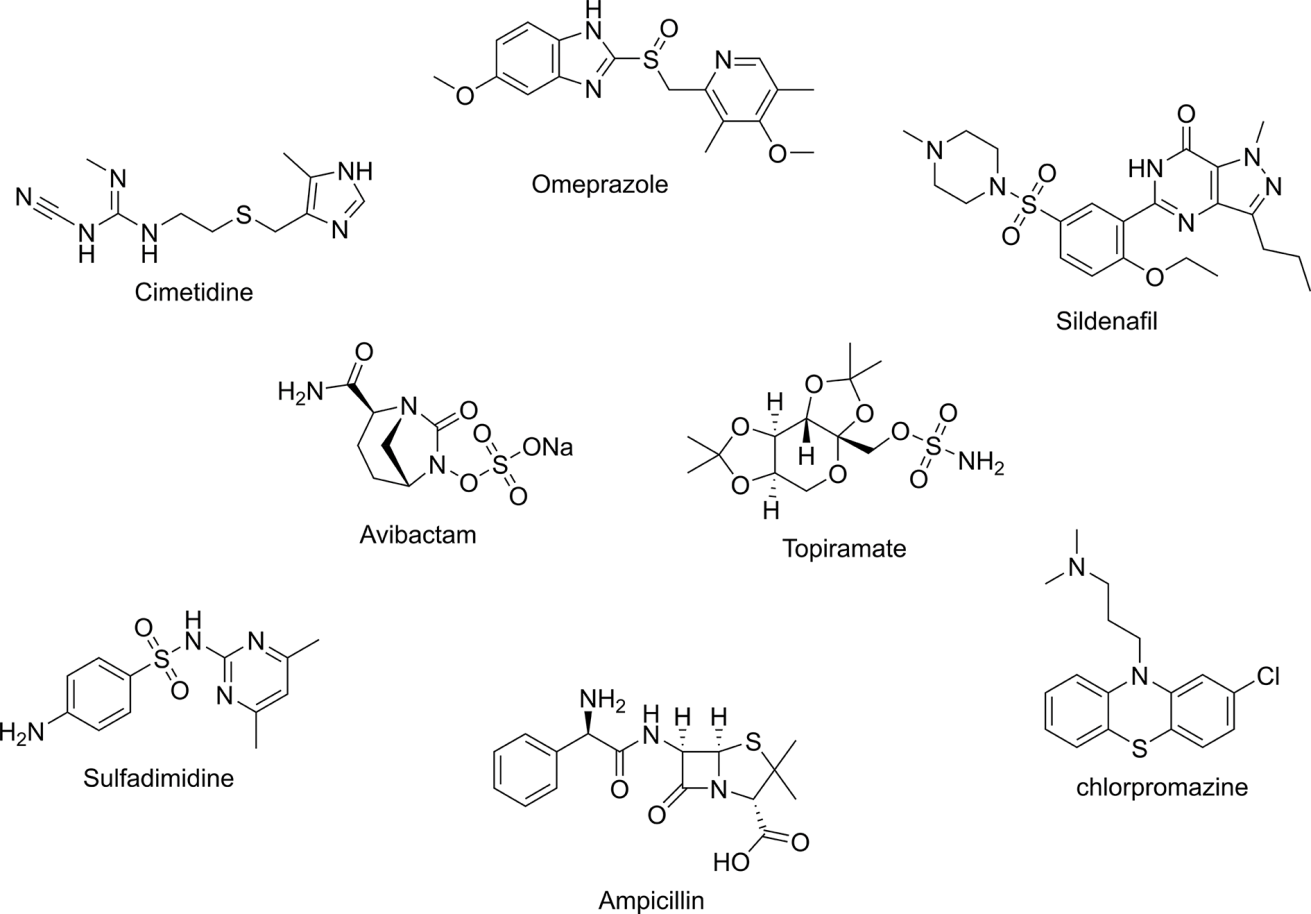
Selected examples of sulfur containing FDA-approved drugs

Sulfur has been recognised as one of the earliest known elements for its therapeutic properties by the ancient Greeks [1]. It is one of the essential components for all living organisms, occurring in the amino acids, methionine and cysteine, and in the vitamins, thiamine and biotin, amongst others [1,2]. Approximately, 250 drugs bearing sulfur-containing functional groups are approved by the FDA, including for hypertension, diabetes mellitus, bacterial infections, migraine, cardiovascular diseases (CVD), neurological disorders, cancer, and human immunodeficiency virus (HIV) (Figure 1) [1–4].

Sulfur is the tenth most abundant element in nature, accounting for approximately 0.03% to 0.06% of the earth’s crust by weight [1]. The sulfur atom exists in multiple oxidation forms, ranging from -2 to +6. Sulfur is present predominantly in the +6-valance state as the sulfate form in the earth's atmosphere.

**Figure 2:**
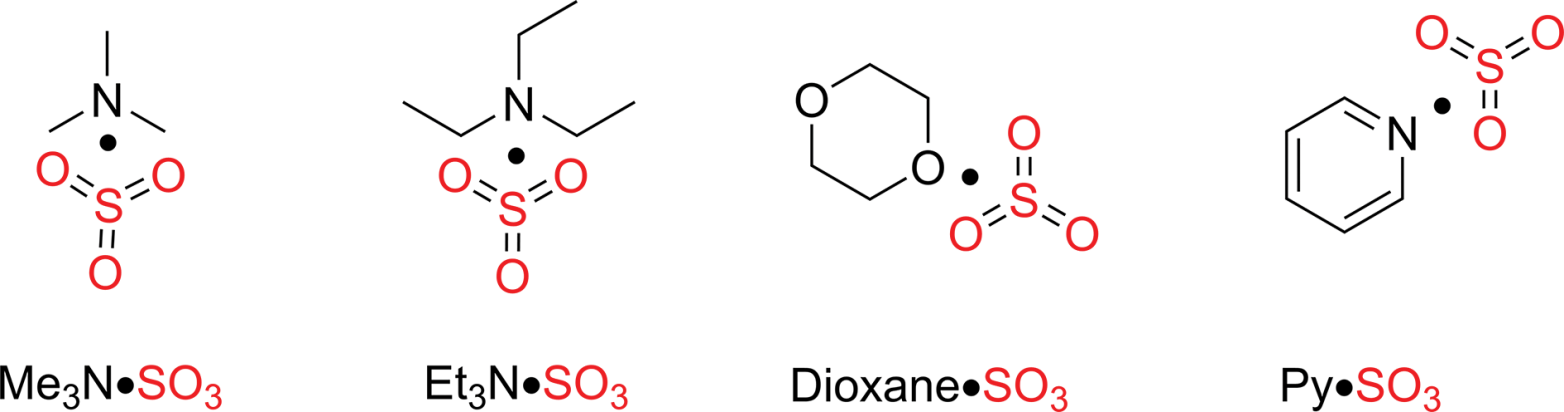
Sulfur trioxide complexes with trimethylamine, triethylamine, dioxane, and pyridine

Sulfur trioxide (SO_3_) is a key precursor to sulfate and present in oleum, a solution of sulfur trioxide (25–65%) in sulfuric acid (H_2_SO_4_) [5]. Moreover, sulfur trioxide is a Lewis acid and engages in reactions with Lewis bases such as trimethylamine (Me_3_N), triethylamine (Et_3_N), dioxane, and pyridine (Py). These reactions result in the formation of sulfur trioxide adducts, which are employed in the sulfation process of various organic substrates, leading to the formation of organosulfate esters (Figure 2) [6].

**Scheme 1:**
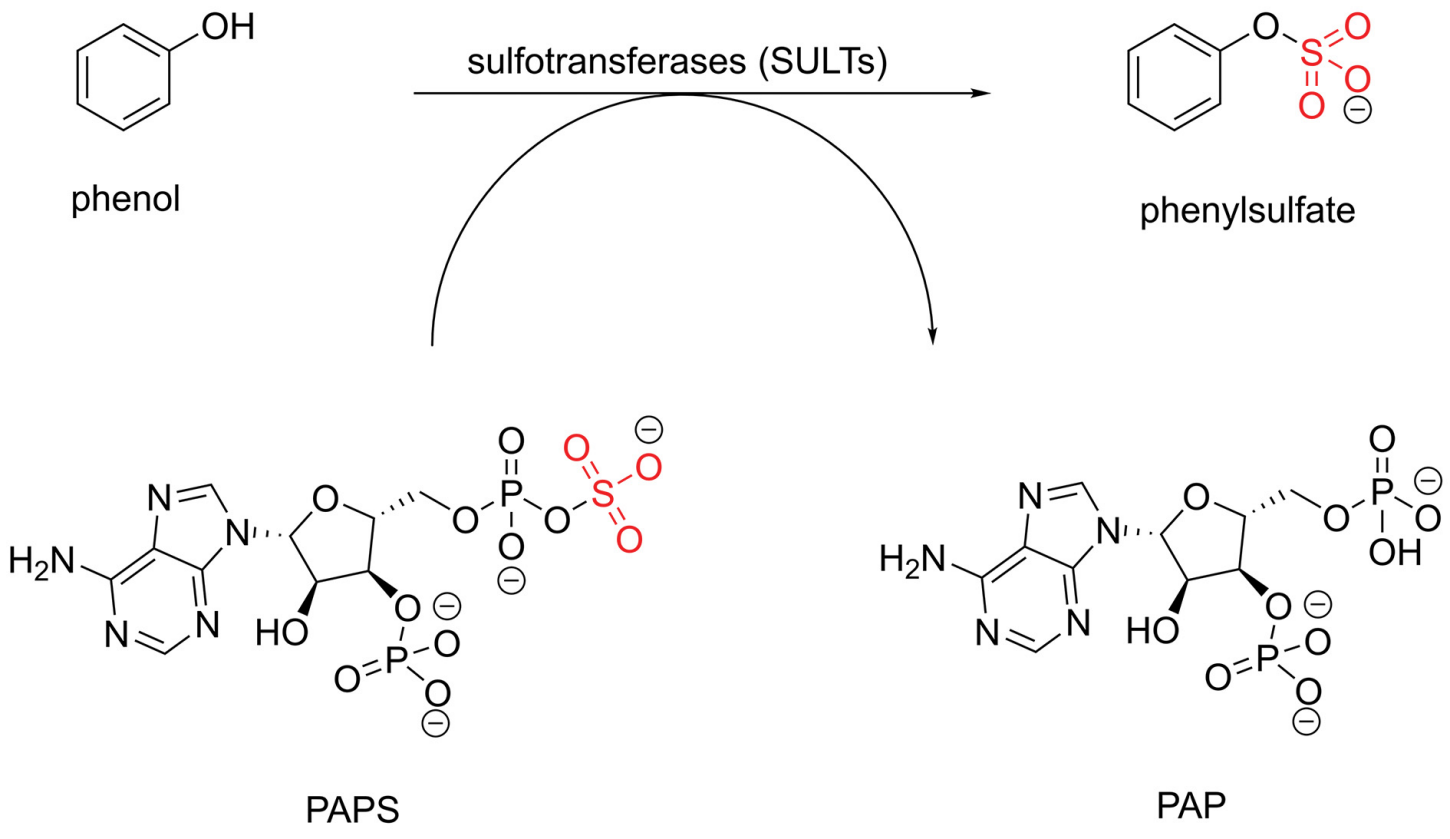
Sulfation of an acceptor substrate (e.g. phenol) mediated by SULT enzyme and PAPS co-factor

Sulfation is an important conjugation reaction during the phase II metabolism of xenobiotics [7]. The sulfation process which mainly occurs in the liver, increases the hydrophilicity of metabolites, facilitating their elimination from the body [8]. Sulfate conjugation usually results in reducing the biological activity of the metabolite [9] but not always. Sulfation can increase the therapeutic activity of certain drugs including minoxidil sulfate, the active form of minoxidil used for the treatment of hypertension and hair loss [10,11]. The sulfation reaction is catalysed by a group of enzymes called sulfotransferases (SULTs), which facilitate the transfer of the sulfate group from 3′-phosphoadenosine 5′-phosphosulfate (PAPS), the universal sulfate donor, to a nucleophilic site of an acceptor molecule such as phenol (Scheme 1) [12–14].

Since the sulfation of the antiseptic, phenol, was discovered in the urine of a patient in 1876 by Eugen Baumann [15] – the role of sulfated small molecules in man has evolved from a detoxification step to critical signalling mechanism. Thus, methods to access these important sulfated biomolecules are critical to understanding the intricacies of the sulfate lifecycle.

### Small molecule chemical sulfation approaches

Organic *O-*sulfates and *N-*sulfamates have several crucial biological applications, ranging from the metabolism of xenobiotics to the downstream signalling of steroidal sulfates in pathological conditions [16]. Anti-coagulant, anti-viral, anti-inflammatory, immunomodulatory, and anti-tumour properties have been associated with sulfated polysaccharides, flavonoids, steroids, and proteins. Heparin and heparan sulfate are examples of glycosaminoglycans (GAGs) that contain sulfate groups which promote ionic molecular interactions with protein ligands and binding at the cellular surface [17]. The incorporation of polar, hydrophilic sulfate groups onto drug-like molecules has facilitated the investigation of novel sulfated biomolecules as potential new therapies [18]. However, the chemical synthesis and purification of sulfated compounds are challenging when one or more sulfate groups are present, due to both anionic crowding, lack of regioselectivity, and poor solubility in organic solvents [19,20]. Successfully sulfated compounds remain sensitive to both acidic and high temperature reaction environments [21]. As a result, the sulfation reaction is often the last step in a synthetic process, which restricts further potential chemical modifications [22].

Given the growing interest in sulfation and the significant biological functions of sulfated molecules, several synthetic approaches to sulfate oxygen, nitrogen, oxime, and phosphate functionalities have been developed. This *Essay* will explore the most commonly encountered and latest chemical sulfation approaches that have been applied to a wide range of small molecules since the last major review in 2010 [23].

### Sulfation using sulfuric acid and related reagents

**Scheme 2:**
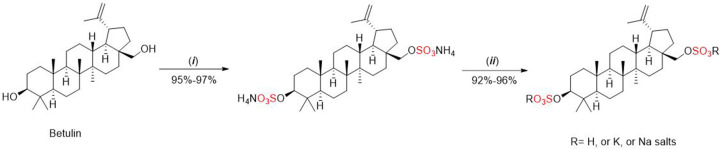
Dual sulfation of betulin with sulfamic acid (NH_2_SO_3_H) Conditions: (i) NH_2_SO_3_H, urea, DMF (60–70°C was 2.0–3.0 h) or 1,4-dioxane (70–75°C, 3.0–3.5 h); (ii) work up with 10% H_2_SO_4_, or 3–5% KOH, or 3–5% NaOH.

Sulfuric acid (H_2_SO_4_) has been employed for the sulfation of (cyclo)alkenes at modest temperature and pressure resulting in (cyclo)alkyl sulfates [6] and the sulfation of polysaccharides and flavonoids [24,25]. The high reactivity of sulfuric acid can be modified via the sulfamic acid (H_2_NSO_3_H) reagent, which has been used for the sulfation of saturated alcohols, carbohydrate, and flavonoids including the naturally occurring triterpenoid betulin*.* Betulin is found in the bark of birch trees and possesses anti-viral, anti-inflammatory, anti-oxidant, and anti-coagulant properties [26]. Betulin was sulfated using the sulfamic acid method in the presence of a urea catalyst and alternatively DMF or 1,4-dioxane as solvent. Dual sulfation of betulin was achieved and isolated as the ammonium, sodium, and potassium salts (Scheme 2).

### Sulfation using chlorosulfonic acid (ClSO_3_H) and its derivatives

**Scheme 3:**
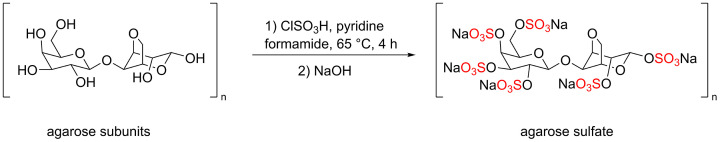
The sulfation reaction of agarose using ClSO_3_H/pyridine method N.B. chlorosulfonic acid was added dropwise to the cold pyridine (0°C) due to the exothermic nature of chlorosulfonic acid.

An alternative approach utilises chlorosulfonic acid, which has been applied for the sulfation of polysaccharides, phenolic acids, and flavonoids amongst others. Agarose sulfate, an example of a sulfated seaweed polysaccharide which was proposed to have an anti-coagulant activity comparable to heparin. Youping and co-workers have reported the formation of agarose sulfate using the chlorosulfonic acid protocol [27]. Agarose polysaccharide was dissolved in formamide solvent followed by the addition of chlorosulfonic acid/pyridine solution and stirred for 4 h at 65°C affording agarose sulfate (Scheme 3).

**Scheme 4:**
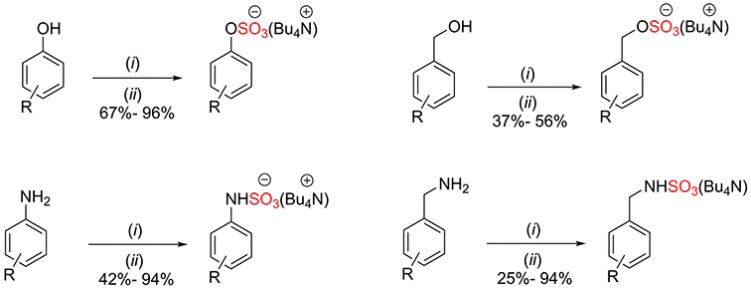
Synthesis of sulfated phenols, benzyl alcohols, anilines, and benzylamine using ClSO_3_H as a sulfation reagent. Followed by the cation exchange with tetrabutylammonium cation. Conditions: (i) ClSO_3_H (5.0 mmol, 1 eq.), triethylamine and DCM, 0°C to r.t., stirred for 1 h. (ii) tetrabutylammonium hydrogen sulfate (4.0 mmol), then extracted into DCM.

A modified version of chlorosulfonic acid-pyridine procedure was implemented using chlorosulfonic acid followed by cation exchange with tetrabutylammonium hydrogensulfate (Bu_4_NHSO_4_) [28,29]. The sulfation reaction of different organic molecules such as phenols, benzyl alcohols, anilines, and benzylamines has been accomplished using this modified protocol (Scheme 4).

### Sulfation using formal sulfur trioxide amine/amide complexes

#### Trimethylamine-sulfur trioxide complex

**Scheme 5:**
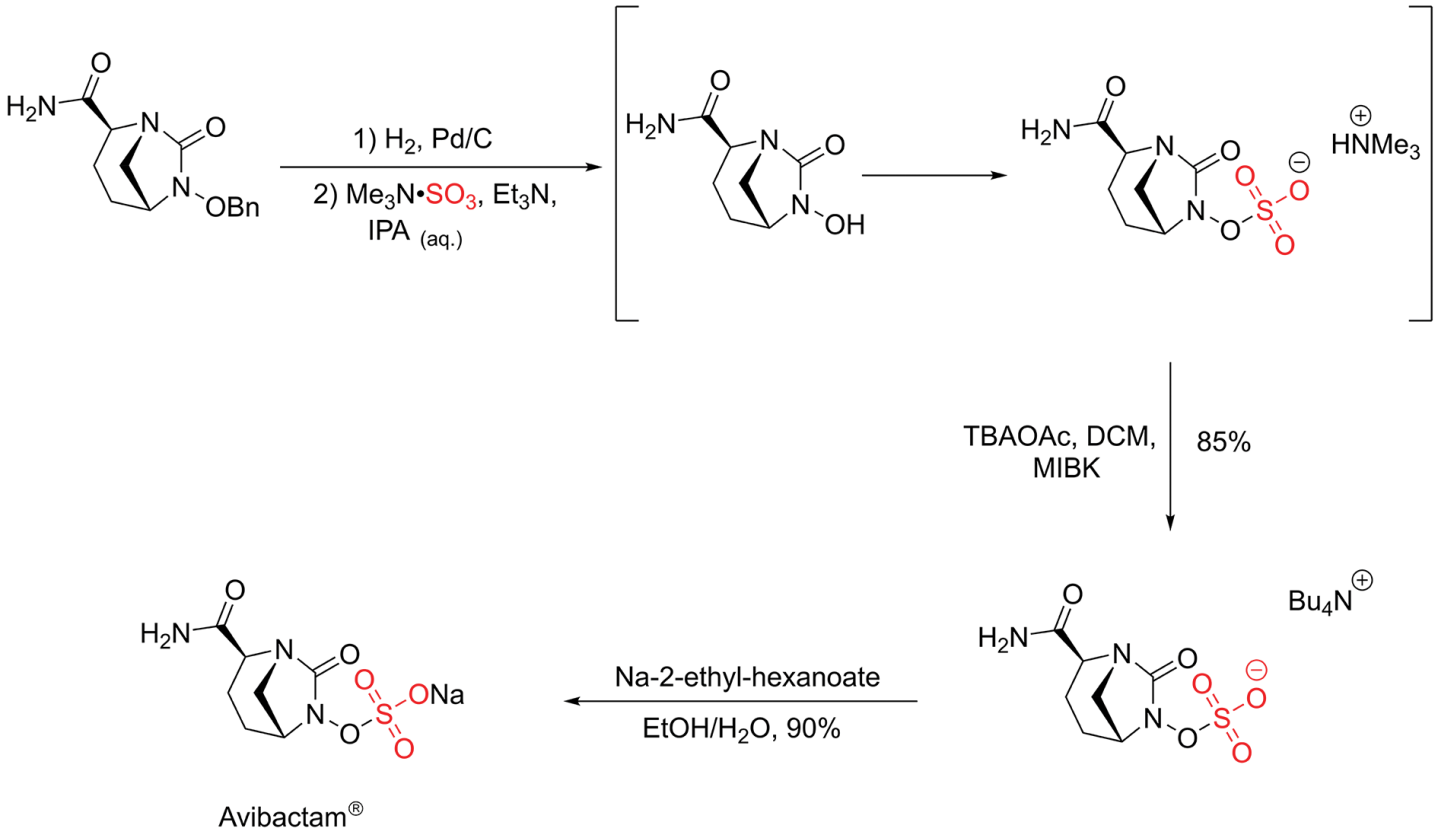
The final sulfation steps during the synthesis of Avibactam®

The sulfation reaction using sulfur trioxide amine/amide complexes is the most used method for alcohol or phenol groups in carbohydrates, flavonoids, steroids, proteins, and related scaffolds. Ball and co-workers have successfully used a sulfur trioxide trimethylamine complex (Me_3_N·SO_3_) and a lipophilic cation-exchange to access Avibactam®, a sulfate containing β-lactamase inhibitor [30]. They reported a simultaneous one-pot deprotection and sulfation reaction of a hydroxylamine intermediate using the Me_3_N·SO_3_ complex. The resulting intermediate was ion exchanged with tetrabutylammonium acetate (TBAOAc) making it more lipophilic and therefore facilitates the extraction of the organosulfate intermediate into DCM. Finally, a lipophilic sodium salt exchange reagent, sodium-2-ethyl-hexanoate (NEH) was added, affording Avibactam® in 90% yield as the sodium salt (Scheme 5).

**Scheme 6:**
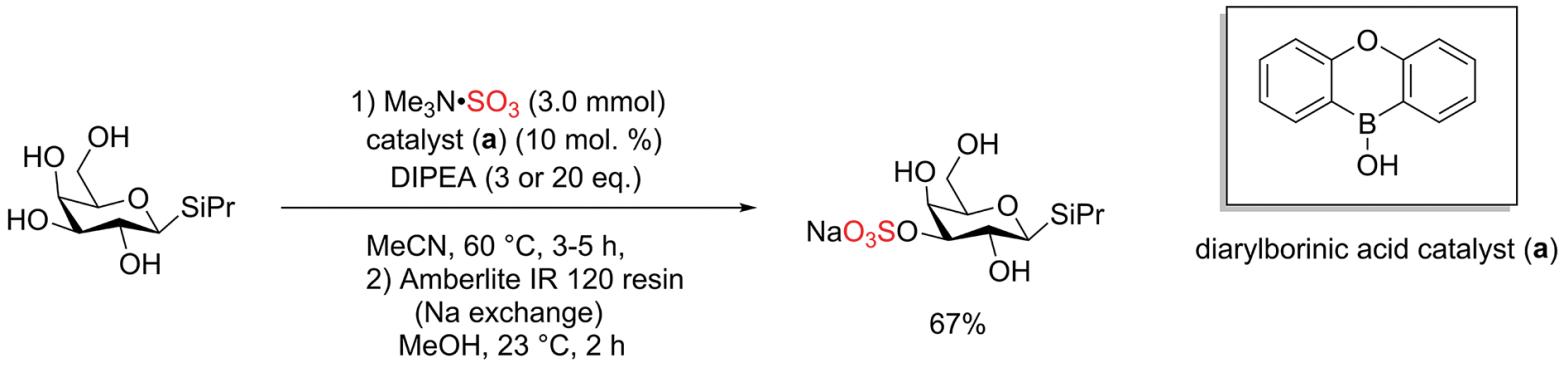
Site selective sulfation of pyranoside scaffolds catalysed by diarylborinic acid (**a**)

The Me_3_N·SO_3_ complex was reported for the site selective sulfation of polysaccharides including pyranoside scaffold [31]. The site selective sulfation of pyranoside scaffolds was carried out under catalytic conditions using diarylborinic acids [32,33]. This method led to the sulfation of different hydroxyl group positions including cis-1,2-diol and 1,3-diol pyranoside derivatives. β-Thioglycoside pyranoside was selected as a model for the site selective sulfation using Me_3_N·SO_3_ and the addition of diarylborinic acid and DIPEA was critical to improve the isolated yield (Scheme 6) [31].

**Scheme 7:**
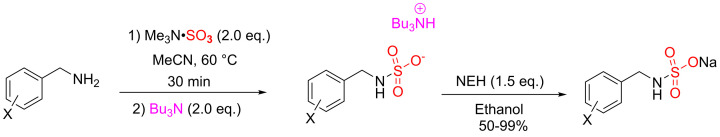
The sulfamation reaction of benzylamine derivatives using Me_3_N**·**SO_3_ in a combination with the lipophilic counterion (Bu_3_N) and a sodium exchange strategy (NEH).

The Me_3_N·SO_3_ complex has also found use in the preparation of sulfamates which contain polar functional groups that are significant to a wide range of biological functions, including viral infection and protein–protein interactions. The sulfamation reaction of benzylamine derivatives were carried out using Me_3_N·SO_3_ complex under a low reaction temperature (30–60°C) affording the corresponding trimethylammonium cation [34]. This was subsequently exchanged with a more lipophilic counterion, tributylamine, then exchanged with a sodium salt (NEH) leading to the formation of sulfamates (Scheme 7) and employed in related examples [35].

**Scheme 8:**
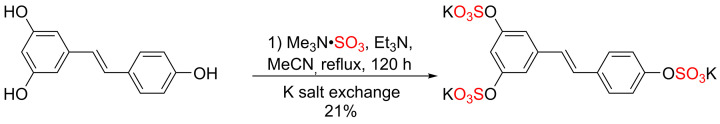
Sulfation reaction of resveratrol using Me_3_N**·**SO_3_ complex

The use of Me_3_N·SO_3_ complex was also reported in the sulfation of resveratrol, a naturally occurring polyphenolic that is found in peanuts, grapes, and berries [36]. It was reported that resveratrol has gained more interest due its important biological applications such as anti-inflammatory, anti-cancer, and anti-oxidant activity. The sulfation reaction of resveratrol was carried out using Me_3_N·SO_3_ complex in the presence of base (Et_3_N) at reflux affording the potassium salt of sulfated resveratrol (Scheme 8).

A summary of the scope and range of sulfated molecules achievable with Me_3_NSO_3_ complex are listed in Table 1.

**Table 1:** Sulfation methods of organic molecules using Me_3_N·SO_3_ complex

Entry	Sulfated substrate	Isolated yield	Reference
1	Avibactam	90%	[30]
2	Pyranoside derivatives	64-97%	[31]
3	Sulfated lactose-derived β-thioglycoside	66%	[31]
4	Sulfated β-galactosylceramide	66%	[31]
5	Benzyl sulfamate derivatives	50-99%	[34]
6	Potassium resveratrol sulfate	21%	[36]

#### Triethylamine sulfur trioxide complex

**Scheme 9:**
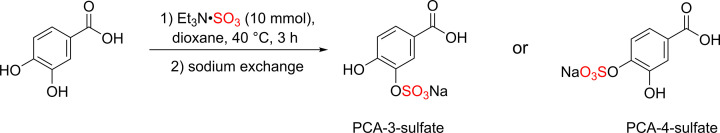
General procedure for sulfation of PCA using Et_3_N**·**SO_3_ affording either PCA-3-sulfate or PCA-4-sulfate

An alternative sulfur trioxide amine complex is triethylamine SO_3_ complex (Et_3_N·SO_3_), which was also used for the sulfation of a wide range of small organic molecules such as flavonoids, polysaccharides, proteins, and steroids. For instance, the sulfation of polyphenolic flavonoids such as protocatechuic acid (PCA), quercetin, and catechin were reported using Et_3_N·SO_3_ complex [37]. PCA sulfate lower the production of interleukin-6 (IL-6) and vascular cell adhesion molecule-1 (VCAM-1), pro-inflammatory cytokine genes that are linked to cardiovascular diseases [38]. Gutierrez-Zetina and co-workers have described the mono-sulfation reaction of PCA using superstoichiometric equivalents of Et_3_N·SO_3_ (10-fold) at 40°C for 3 h. As a result, two compounds were isolated as PCA-3-sulfate and PCA-4-sulfate, respectively (Scheme 9) [37].

The mono-sulfate analogues of quercetin and catechin were also prepared following the mono-sulfation reaction procedure used with PCA employing Et_3_N·SO_3_ as a source of the sulfate group [39]. Correia-da-Silva and co-workers have investigated the synthesis of persulfated compounds which can be used as an anticoagulant and antiplatelet agents for the treatment of thrombosis [40]. Sulfation of different polyphenolic molecules such as gallic acid, 4-methyl 7-hydroxycoumarin 7-ß-D-glucopyranoside, and ascorbic acid was afforded using Et_3_N·SO_3_ (2–8 eq./OH group) in dimethylacetamide (DMA) at 65°C for 24 h reaction duration. A summary of the scope and range of sulfated molecules achievable with Et_3_NSO_3_ complex are listed in Table 2.

**Table 2:** Sulfation methods of organic molecules using Et_3_N·SO_3_ complex

Entry	Sulfated substrate	Isolated yield	Reference
1	PCA-3-sulfate	N.R.	[37]
2	Quercetin 3-sulfate	N.R.	[39]
3	Sulfated gallic acid	36%	[40]
4	Sulfated 4-methyl 7-hydroxycoumarin	47%	[40]
5	Sulfated ascorbic acid	7%	[40]

N.R. = not reported

During the writing of this Essay, single crystal X-ray crystallography confirmed that triethylamine-sulfur trioxide complex also exists as triethylsulfoammonium betaine (TESAB) not a dative bond adduct [41].

#### Tributylsulfoammonium betaine (TBSAB)

**Scheme 10:**
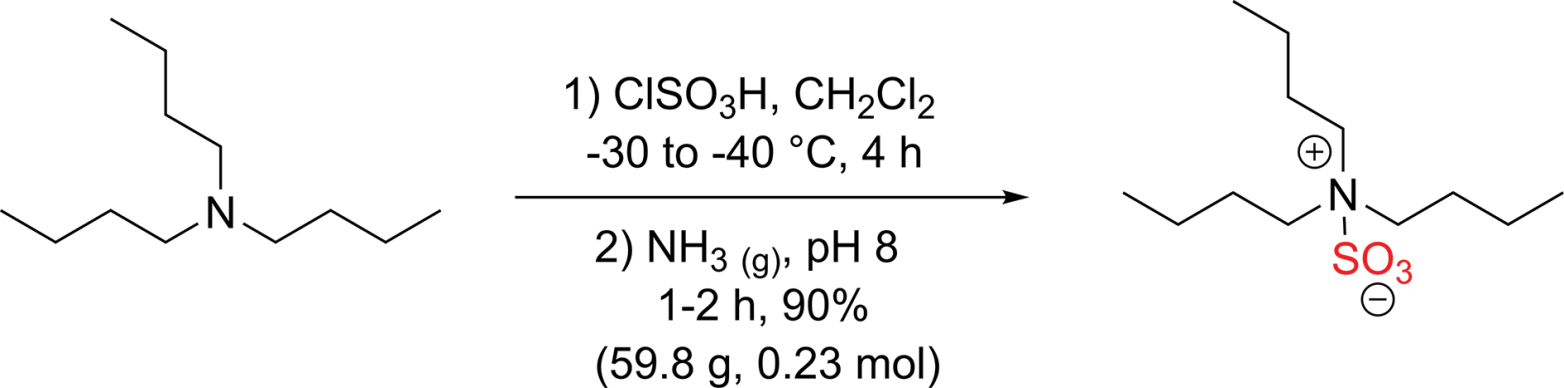
Synthesis of sulfur trioxide tributylamine complex (TBSAB)

A novel sulfating reagent was recently developed by Gill and co-workers, tributylsulfoammonium betaine (TBSAB) (Scheme 10) [42].

**Scheme 11:**
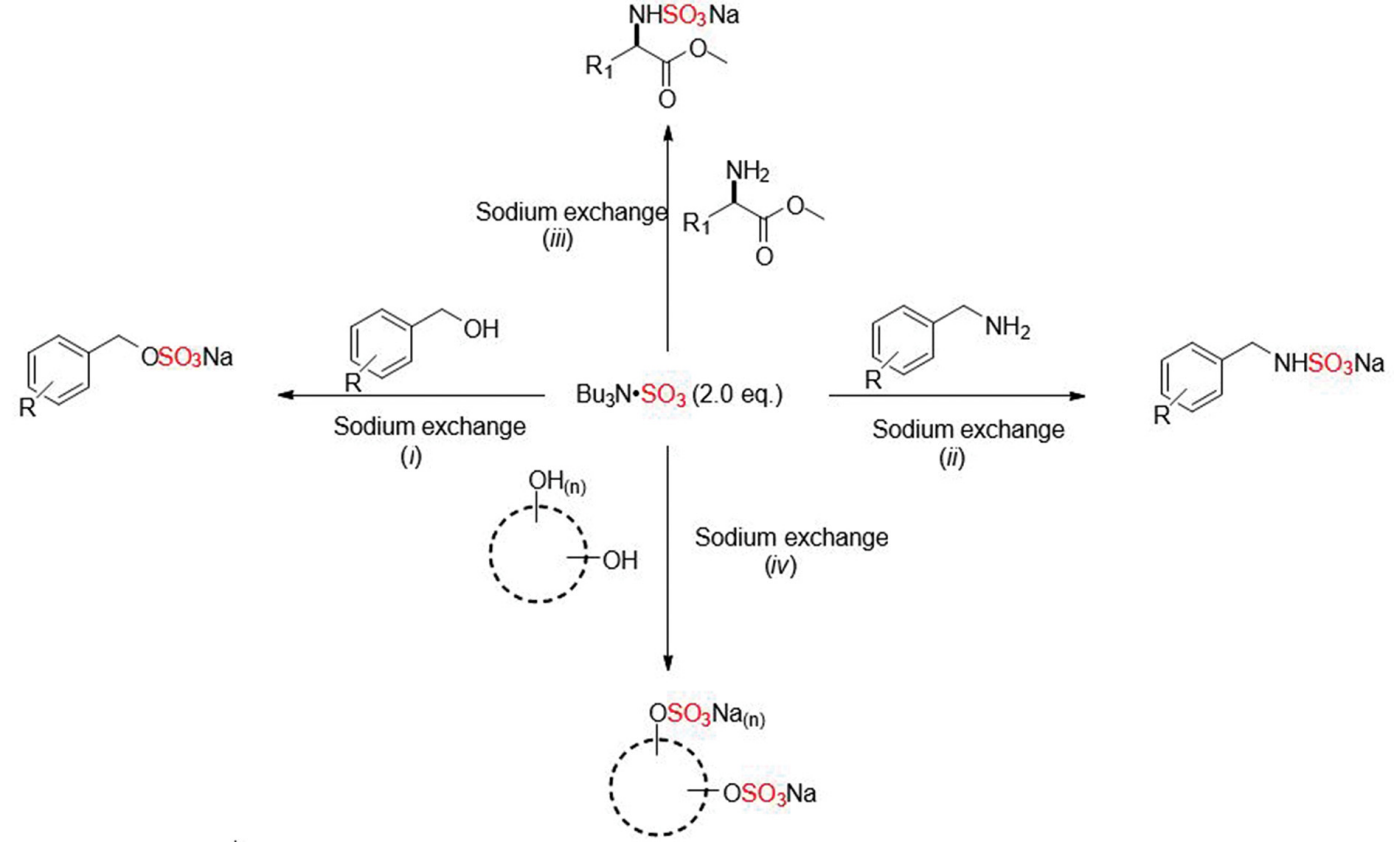
General sulfation synthesis of selected organic scaffolds using the all-in-one reagent, Bu _3_N·SO_3_ Conditions: (i) Bu _3_N·SO_3_ (2.0 eq.), MeCN, 90°C, up to 3 h, then NEH or NaI exchange. (ii) Bu_3_N·SO_3_ (2.0 eq.), MeCN, 30°C, up to 1 h, then NaI exchange. (iii) Bu_3_N·SO_3_ (2.0 eq.), MeCN, r.t., up to 18 h, then NaI exchange. (iv) Bu_3_N·SO_3_ (≥ 2.0 eq.), MeCN, 90°C, up to 12 h, then NEH or NaI exchange. R_1_ = amino acid side chain.

TBSAB provides a simplified purification and isolation of sulfated molecules due to the greater lipophilicity profile of the corresponding tributylammonium intermediate (log_10_*P* = 4.01) [43]. The scope of the TBSAB reagent is demonstrated in Scheme 11.

TBSAB was used for the sulfation and sulfamation of a wide range of benzyl alcohols, benzylamines, amino acids, and carbohydrates [44,45]. TBSAB has also been used for the chemoselective sulfation of steroids affording the corresponding sulfated steroid molecules such as, estrone sulfate, pregnenolone sulfate, and pregnanediol sulfate [46]. TBSAB has also found use as a reagent to install aniline *N*-sulfamates and ylideneamino sulfates prior to their intermolecular rearrangement [47,48]. A summary of the scope and range of sulfated molecules achievable with Bu_3_NSO_3_ complex are listed in Table 3.

**Table 3:** Sulfation methods of selected biomolecules using Bu_3_N·SO_3_ (TBSAB) complex

Entry	Sulfated substrate	Isolated yield	Reference
1	Sulfated glycerol	92%	[42]
2	Sulfated 2-hydroxyphenyl ethanol	74%	[42]
3	*L*-phenylalanine methyl ester sulfamate	60%	[44]
4	*L*-cysteine methyl ester sulfamate	50%	[44]
5	Sulfated glycomimetic C3	76%	[42,45]
6	Estradiol sulfate	84%	[42,46]
7	pregnenolone sulfate	98%	[46]

#### Pyridine-sulfur trioxide and dimethylformamide-sulfur trioxide complexes

**Figure 3:**
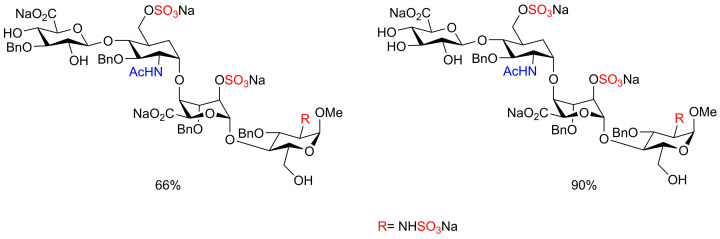
Sulfated tetrasaccharide examples prepared by using the Py·SO_3_ complex

**Scheme 12:**
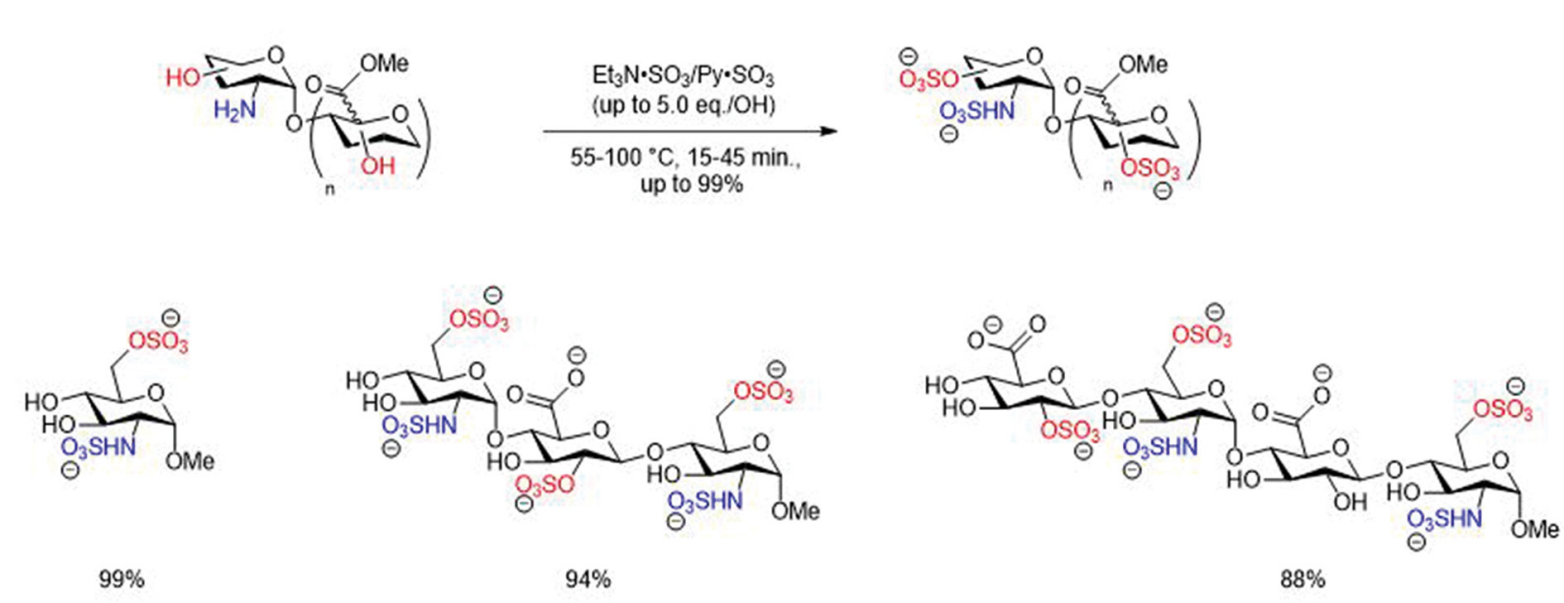
General preparation of sulfated HP/HS like saccharides using microwave-assisted approach with Et_3_N·SO_3_ and Py·SO_3_ sulfating reagents

Alternative sulfur trioxide complexes involving Py·SO_3_ and DMF·SO_3_ were used for the sulfation of polysaccharides and polyphenolic flavonoids [49]. Sun and co-workers reported the installation of sulfates and sulfamate moieties onto protected heparan sulfate oligosaccharide derivatives using Py·SO_3_ complex. The preparation of a sulfated and sulfamated tetrasaccharide substrate was achieved despite multiple reaction steps and the use of several protecting groups which added complexity to the sulfation process (Figure 3) [50]. Xu and co-workers have reported the use of an efficient microwave-assisted approach for the simultaneous *O,N*-sulfation of heparin and heparan sulfate saccharides despite the multi-step reaction synthesis including protection/deprotection steps. In this approach, both Et_3_N·SO_3_ and Py·SO_3_ sulfating agents were employed in a solvent mixture of Et_3_N/pyridine at between 55–100°C for 15–45 min. This approach was appropriate to install sulfate/sulfamate moieties onto mono-, di-, tri- and tetra-saccharides in a short reaction time and good to excellent isolated yields (Scheme 12) [51].

**Scheme 13:**
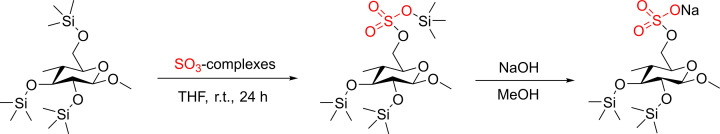
The regioselective sulfation of trimethylsilyl cellulose (TMSC) using sulfur trioxide complexes

Cellulose sulfate is an example of sulfated polysaccharide which has been studied for its potential biological and pharmaceutical applications such as anti-coagulant, anti-microbial, anti-oxidant, and used in drug delivery systems. Richter and co-workers have described the regioselective sulfation of the trimethylsilyl cellulose (TMSC) using sulfur trioxide complexes including Py·SO_3_, DMF·SO_3_, and Et_3_N·SO_3_ [52]. The resulting intermediate was treated with sodium hydroxide yielding the final desired sodium cellulose sulfate as the sodium salt (Scheme 13).

**Figure 4:**
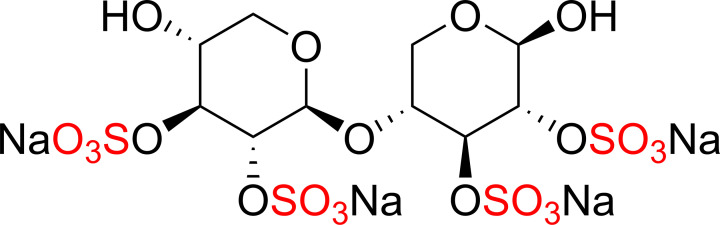
The structure of pentosan polysulfate sodium (Elmiron®) which was approved for the treatment of interstitial cystitis

Malins and co-workers have reported the use of Py·SO_3_ and DMF·SO_3_ complexes for the preparation of sulfated xylooligosaccharides that could be a promising therapeutic agent similar to the known exemplar, pentosan polysulfate (PPS) [53]. Pentosan polysulfate is a semi-synthetic polysulfated xylan that is related to glycosaminoglycans (GAGs) containing β-1→4-linked xylooligosaccharides and was approved for the treatment of interstitial cystitis (inflammation of bladder) (Figure 4) [54].

**Scheme 14:**
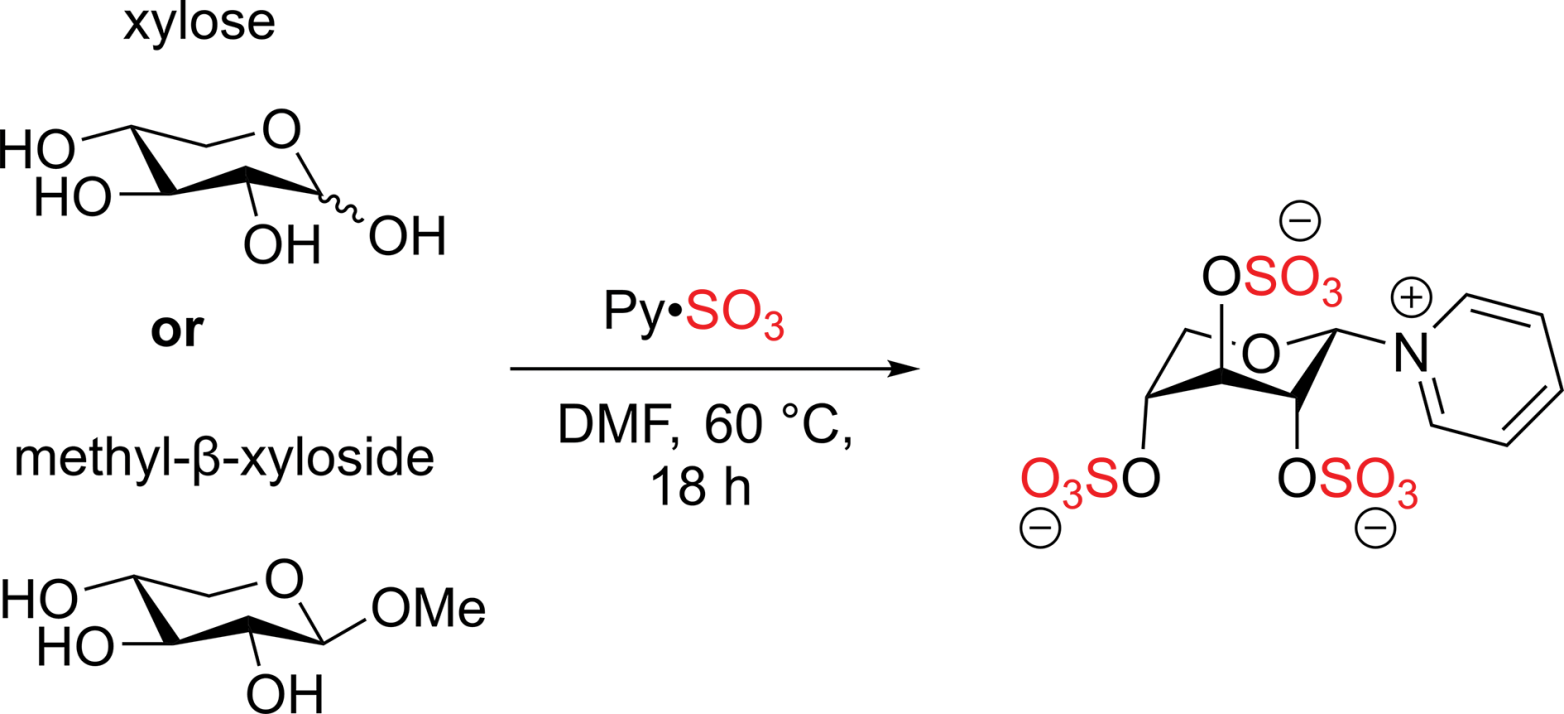
The sulfation reaction of xylan derivatives using Py·SO_3_ complex

An initial attempt for the sulfation of xylooligosaccharides was examined on different substrates such as xylose, methyl-β-xyloside, and xylobiose used Py·SO_3_ [53]. Unfortunately, the β-1→4 linkage of xylan derivatives was cleaved by the nucleophilic addition of pyridine (Scheme 14) [55].

**Scheme 15:**
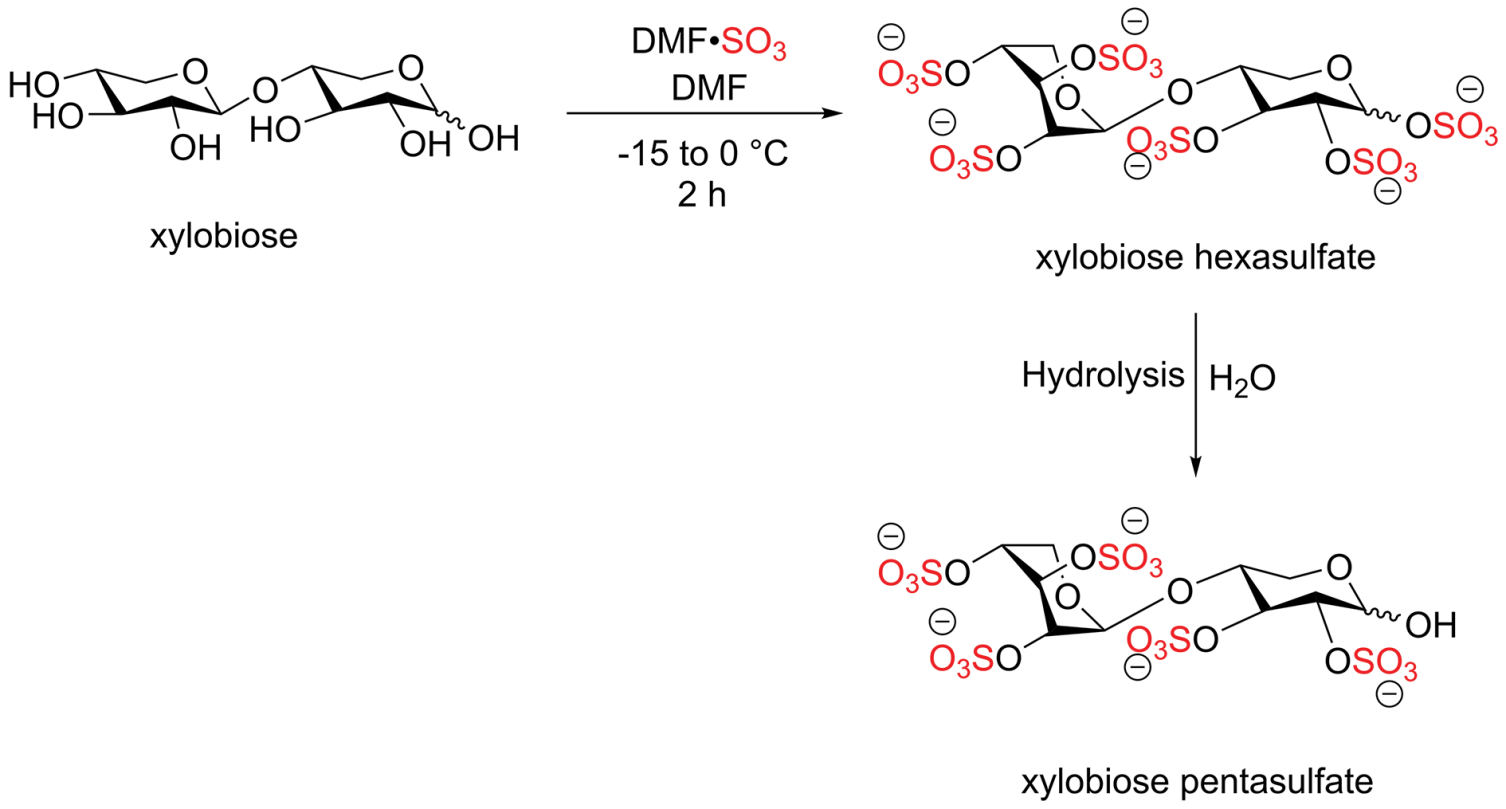
The sulfation reaction of xylobiose using DMF·SO_3_ complex affording xylobiose hexasulfate and pentasulfate

Due to the previous unsuccessful attempt with Py·SO_3_, DMF·SO_3_ complex was used for the sulfation of xylobiose [53]. The sulfation of xylobiose with DMF·SO_3_ afforded xylobiose hexasulfate and xylobiose pentasulfate, a reduced sugar, which was formed via hydrolysis of the hexasulfate product (Scheme 15).

**Scheme 16:**
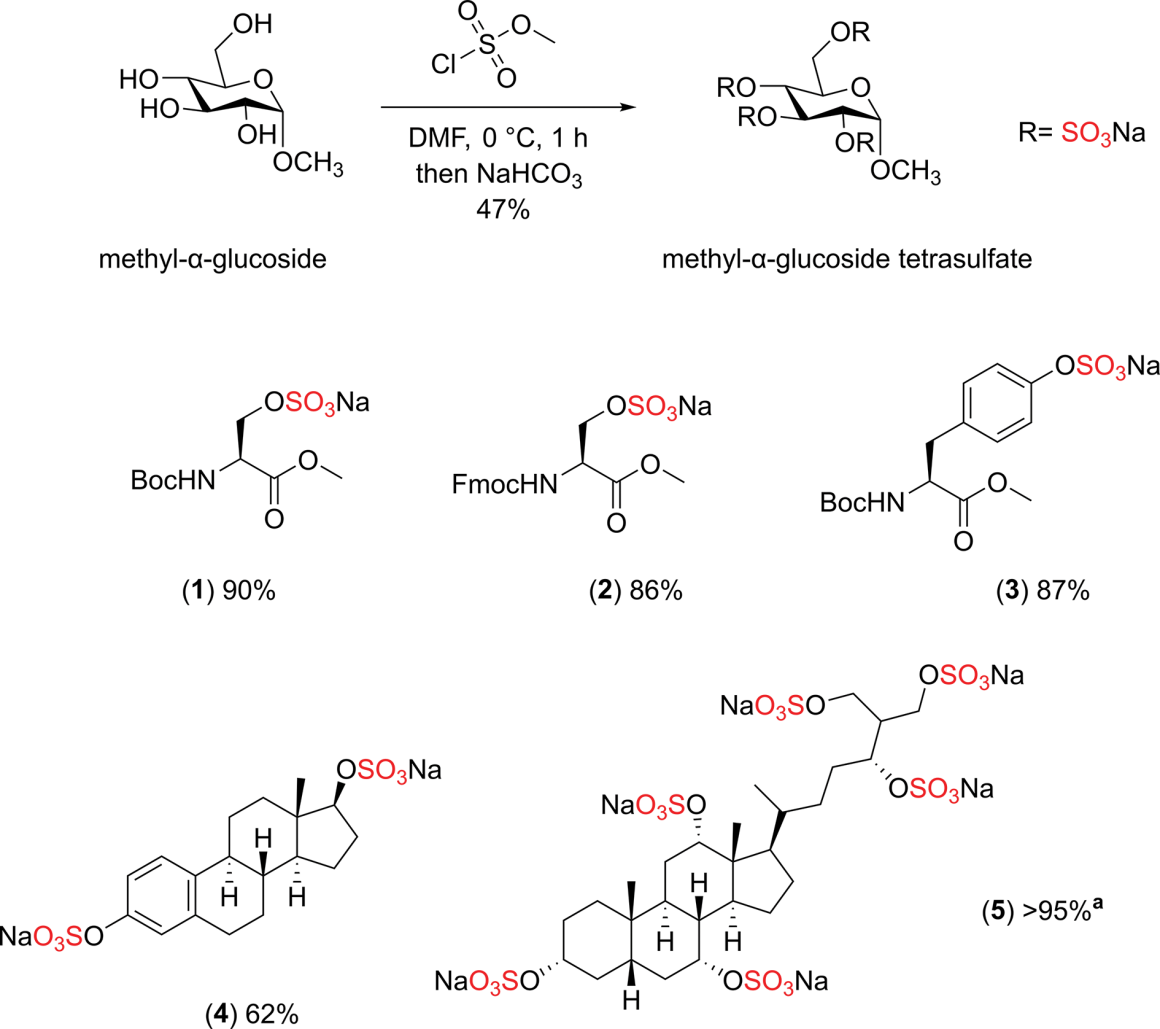
The sulfation reaction of methyl-α-glucoside using methyl chlorosulfate - DMF protocol The scope of sulfated small molecules with methyl chlorosulfate, sodium Boc-Serine methyl ester sulfate (**1**), sodium Fmoc-*L*-Serine methyl ester sulfate (**2**), sodium Boc-tyrosine methyl ester sulfate (**3**), sodium β-estradiol disulfate (**4**), and sodium scymnol persulfate (**5**). **^a^** percentage conversion calculated by ^1^H NMR spectroscopy.

Malins. has also reported an *in situ* synthesis of DMF·SO_3_ complex using the strategy of the addition of methyl chlorosulfate to DMF at 0°C. This reaction is exothermic leading to the formation of methylchloride as a side product [53]. This protocol was initially investigated on the methyl-α-glucoside following the optimised conditions, affording the methyl-α-glucoside tetrasulfate in 47% isolated yield. This protocol was further explored on a wide range of small molecules including amino acids, disaccharide, steroids, and acid-sensitive substrates (Scheme 16).

### Alternative reactivity of sulfur-based alkylating agents

**Scheme 17:**
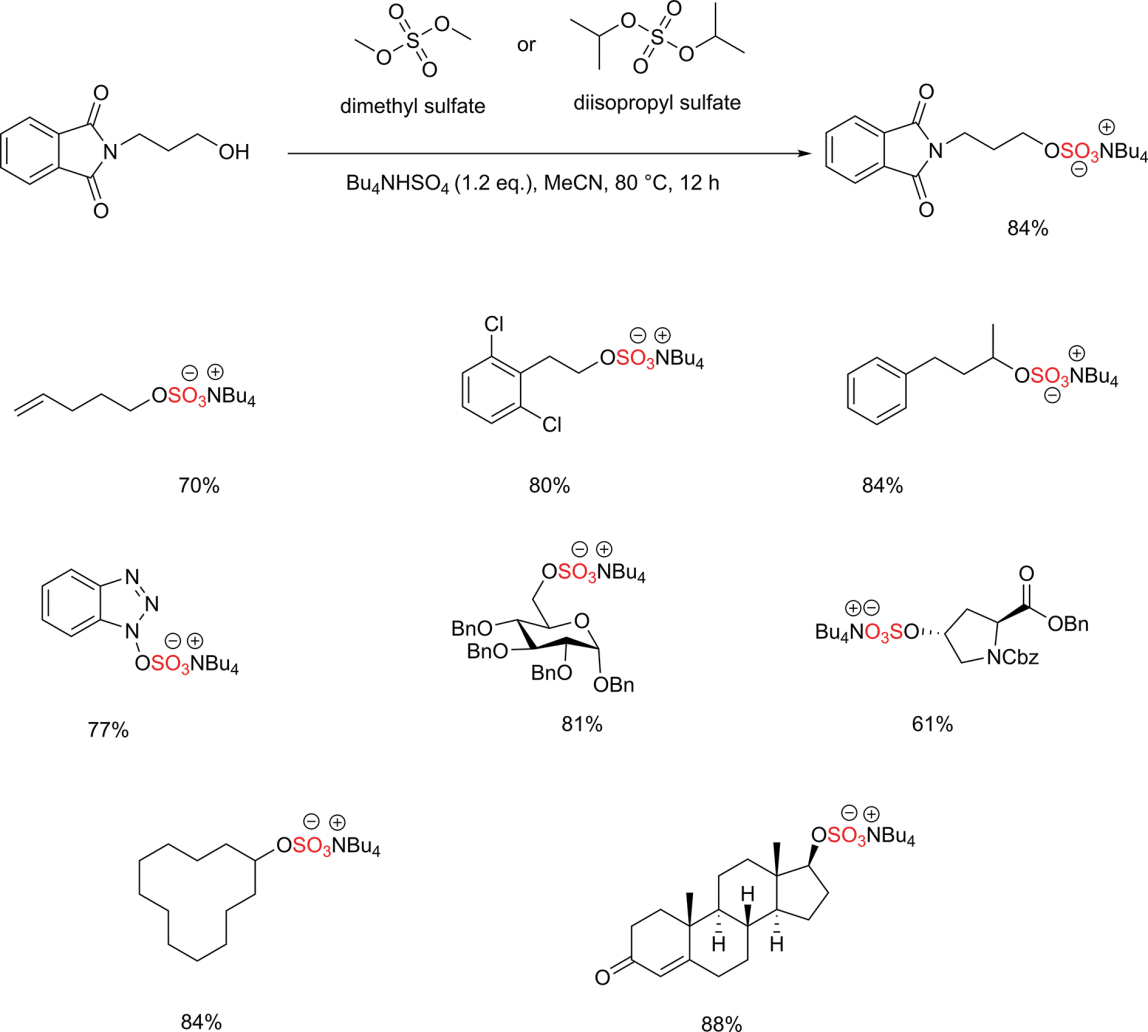
The *O*-sulfation of a broad range of molecules using DMS/DPS and tetrabutylammonium bisulfate (Bu_4_NHSO_4_)

During the writing of this *Essay*, Yue and co-workers have reported an innovative strategy for the *O*-sulfation using dimethyl sulfate (DMS) and diisopropyl sulfate (DPS) [56]. This strategy requires tetrabutylammonium bisulfate (Bu_4_NHSO_4_) [57] to activate the dialkyl sulfates, which facilitates the sulfation of a wide range of functional groups, including alcohols, phenols, and oxime containing substrates. Furthermore, tetrabutylammonium bisulfate improves the solubility of the sulfated products in organic solvent. This investigation was initiated with the reaction of 3-phthalimido-1-propanol and DMS affording the tetrabutylammonium 3-phthalimido-1-propanol sulfate in 84% isolated yield (Scheme 17).

#### Sulfation using sulfur (VI) fluoride exchange (SuFEx) reaction

**Scheme 18:**
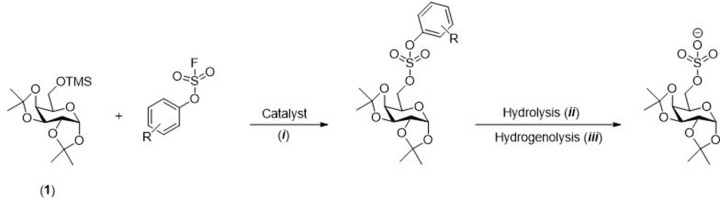
General *O*-sulfation of protected galactopyranose General *O*-sulfation of protected galactopyranose (**1**) using SuFEx strategy. Conditions: (i) DBU, MeCN, 2 h, r.t.; (ii) 5 M sodium methoxide, 1 h, r.t.; (iii) Pd(OH)_2_/C and H_2_, buffer solution (MeCN/MeOH/PBS) 2:2:1, 2 h, r.t.

**Figure 5:**
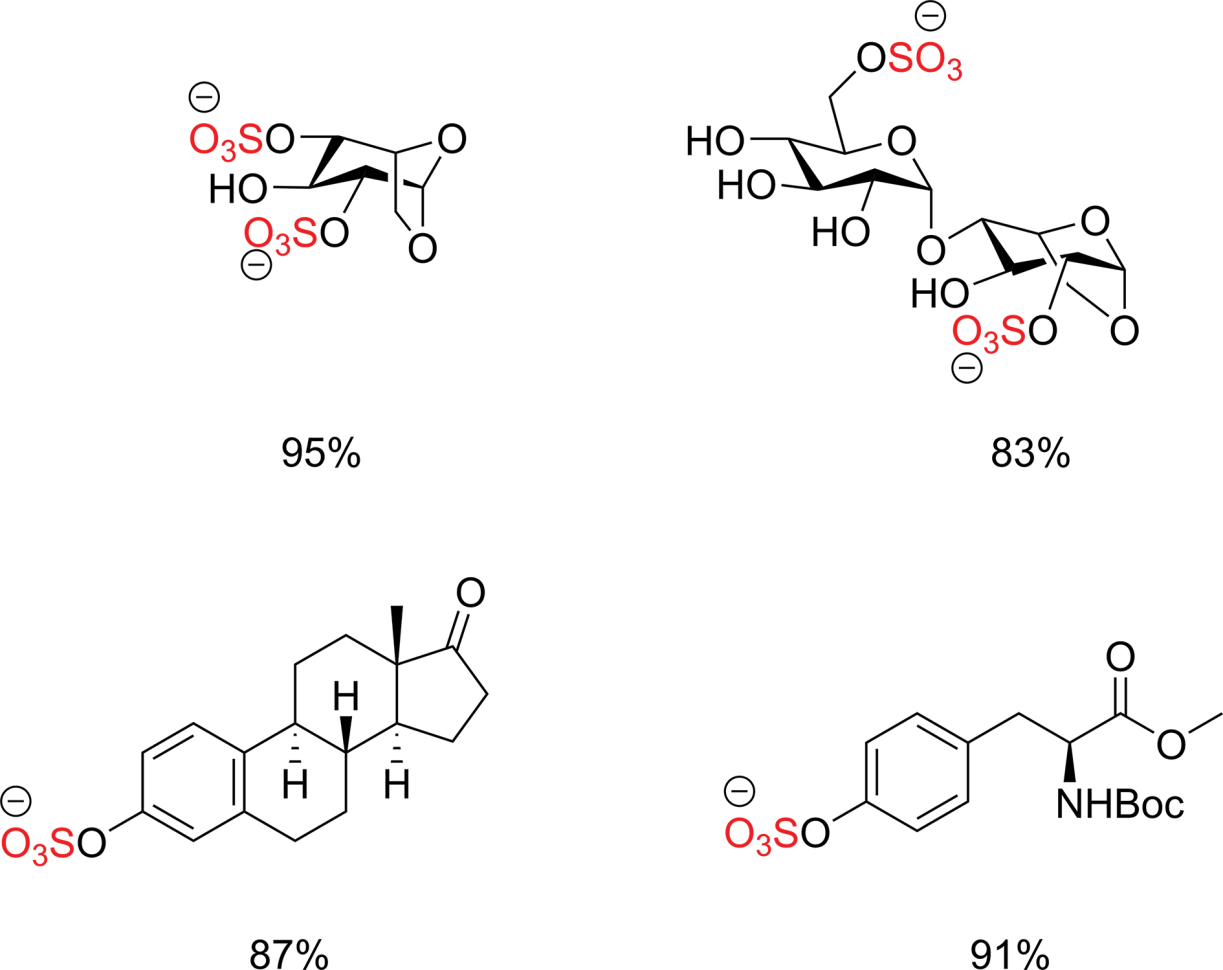
Scope of sulfated biomolecules using the SuFEx approach

Recently, early stage *O*-sulfation reaction between aryl fluorosulfates and silyl ethers (Scheme 18) was reported leading to the formation of sulfuric acid diesters, which are subsequently deprotected to the target sulfates via a hydrogenolysis step [58]. This strategy was applied to a wide range of small molecules such as monosaccharides, disaccharides, an amino acid, and a steroid by Liu and co-workers (Figure 5) [58].

#### Protection/deprotection approaches

**Scheme 19:**
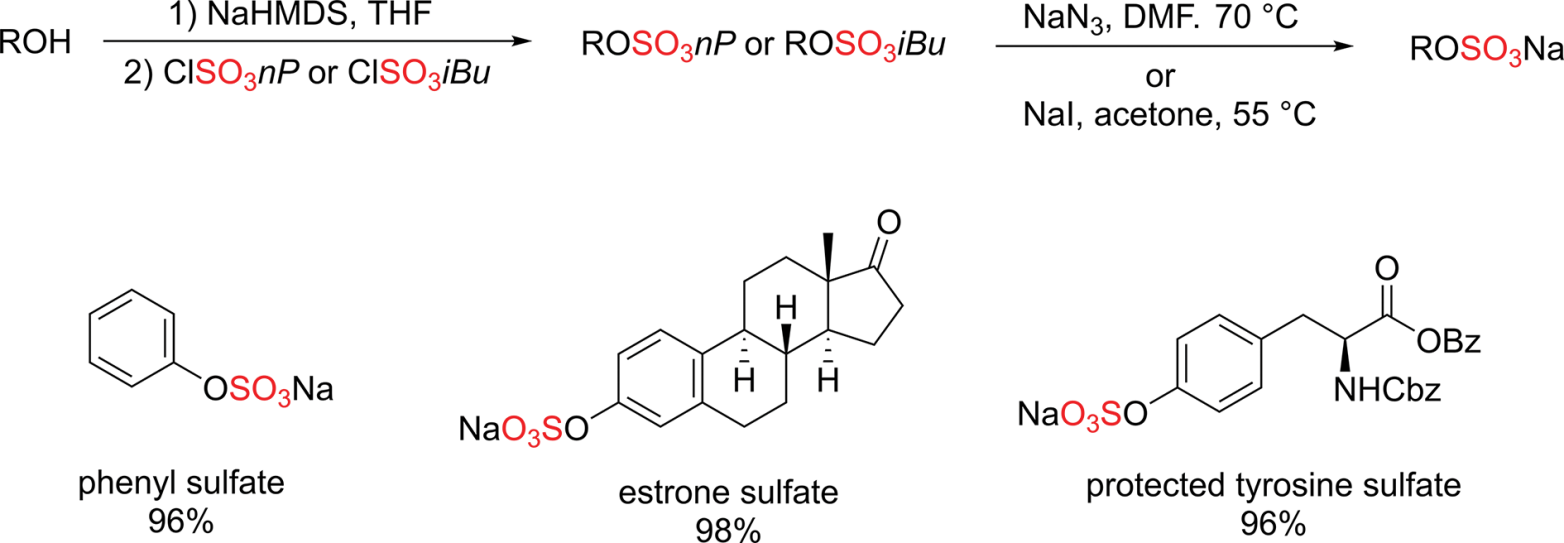
Protection/deprotection method using the protected sulfate neopentyl or isobutyl esters Followed by the deprotection reaction with NaN_3_ for the removal of neopentyl (*nP*) protecting group and sodium iodide (NaI) for the removal of isobutyl (*iBu*) protecting group affording the final desired sulfates.

Despite the effectiveness of direct sulfation methods, the formation of complex sulfated molecules may be hampered by the poor solubility of sulfated molecules in organic solvents, stability issues, and purification challenges of sulfated molecules [23]. As a result, there was a growing interest in developing protection/deprotection strategy which involves the incorporation of sulfate group(s) in a masked form into the target scaffold followed by a deprotection step affording the final sulfate molecules [23,59]. Simpson and co-workers have used alkyl protecting groups such as isobutyl (iBu) and neopentyl (nP) groups for the preparation of sulfate monoesters [59]. In this method, phenol or alcohol containing substrates were initially treated with a strong base such as sodium hydride (NaH) or sodium hexamethyldisilazide (NaHMDS) at −75°C followed by the addition of isobutyl or neopentyl chlorosulfate affording the desired protected sulfate monoesters. Subsequent deprotection step using sodium azide or sodium iodide affording the desired sulfates in most cases (Scheme 19) [59].

## Conclusions and outlook

Sulfation is one of the most important modifications that occur to a wide range of small biomolecules including polysaccharides, proteins, flavonoids, and steroids. The incorporation of a sulfate moiety to an appropriate substrate, results in an increase of the substrate’s hydrophilicity and therefore facilitate its elimination from the body. However, sulfated scaffolds such as polysaccharides, proteins, flavonoids, and steroids have significant biological and pharmacological roles such as cell signalling, modulation of immune and inflammation response, anti-coagulation, anti-atherosclerosis, and anti-adhesive properties. It is imperative that sulfated molecules can be easily prepared to further extend the biological understanding of the role of sulfate in the body. An emerging area of importance for analytical chemistry but outside the scope of this *Essay* is enzymatic modification for the synthesis of low molecular weight heparins, protein and carbohydrate sulfation [60–63].

**Figure 6:**
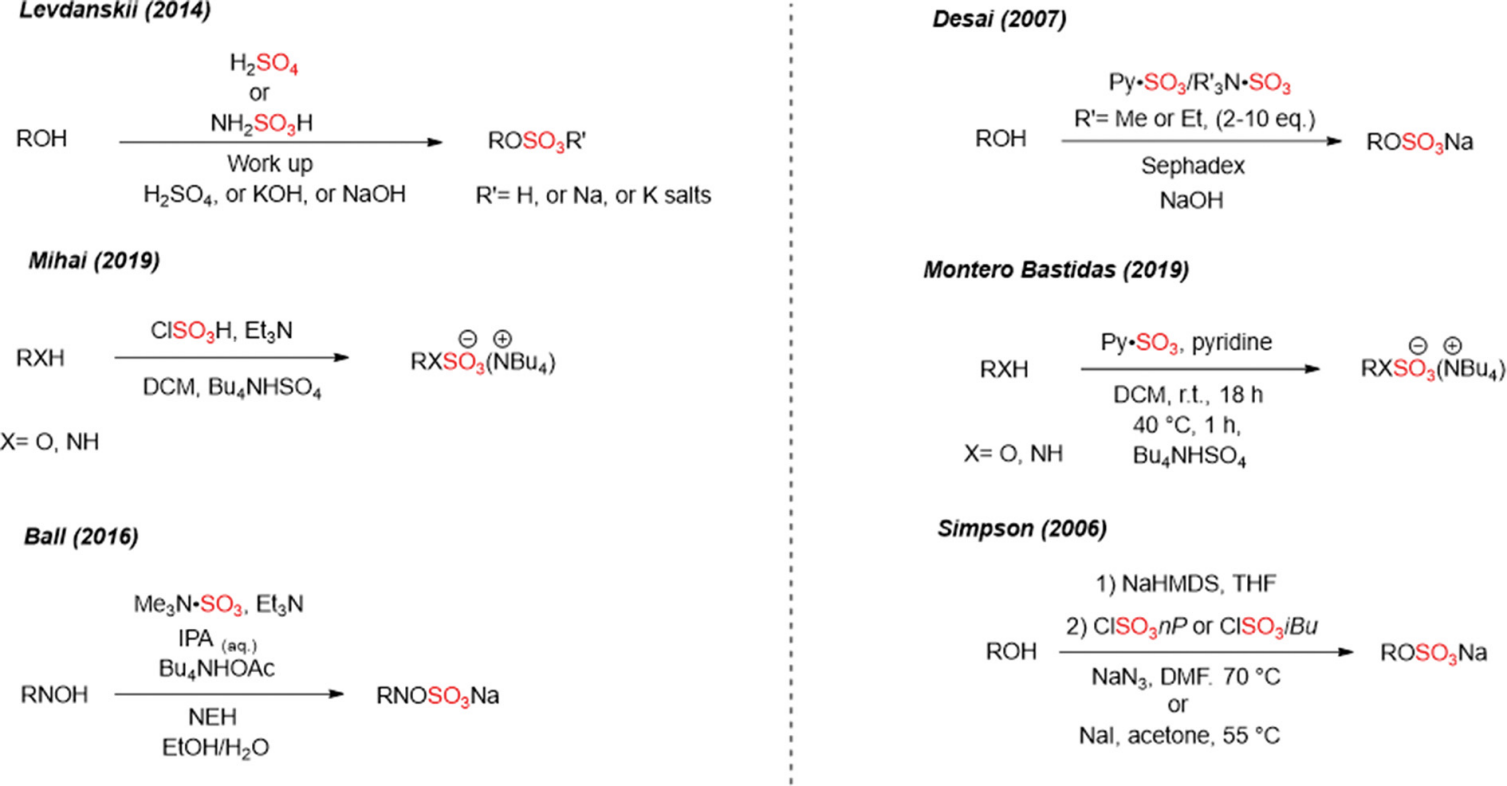
Previous conventional sulfation methods Including sulfuric acid/sulfamic acid, chlorosulfonic acid, sulfur trioxide amine complexes, and the protection/deprotection methods which suffer from some issues such as multiple-purification steps reactions, toxicity issues (e.g. pyridine contamination), purification challenges, stoichiometric excess of reagents which leads to increase of a reaction cost, and intrinsic stability issues of both the reagent and product.

This *Essay* summarised the most encountered chemical sulfation methods of small molecules (Figure 6). Traditional sulfation reactions have relied on sulfuric acid derivatives. Sulfation reaction using sulfur trioxide amine/amide complexes was the most used method for alcoholic or phenolic groups in carbohydrates, steroids, proteins, and aliphatic or alicyclic scaffolds. Despite the effectiveness of these methods, they suffer from some issues such as multiple-purification steps reactions, toxicity issues (e.g. pyridine contamination), purification challenges, stoichiometric excess of reagents which leads to increase of a reaction cost, and intrinsic stability issues of both the reagent and product.

**Figure 7:**
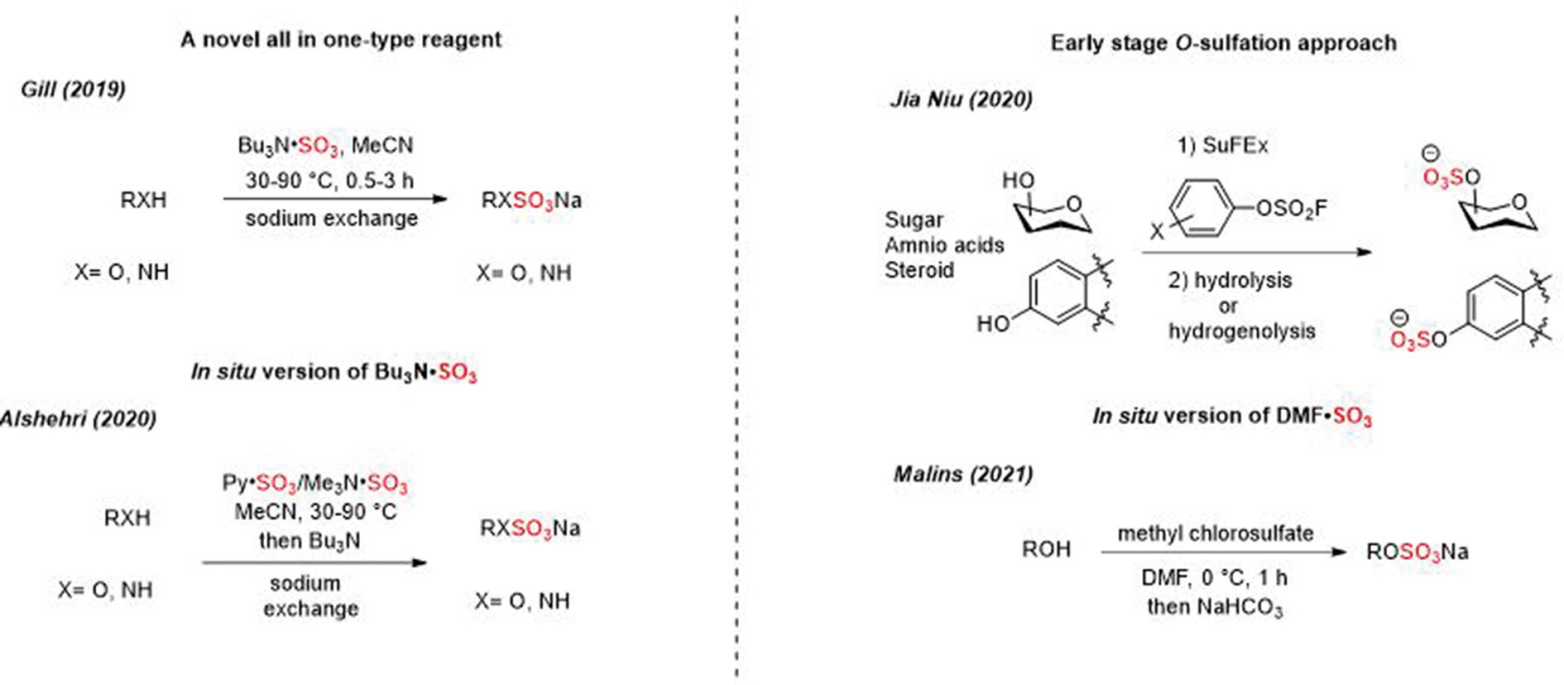
Recent chemical sulfation methods including the novel sulfating reagent, TBSAB-Bu_3_N·SO_3_, an *in situ* version of Bu_3_N·SO_3_, *O*-sulfation stage using SuFEx, and the* in situ* version of DMF·SO_3_.

Recent advances (Figure 7) including SuFEx, the *in situ* reagent approach and TBSAB show the widespread appeal of novel sulfating approaches that will enable a larger exploration of the field in the years to come by simplifying the purification and isolation to access bespoke sulfated small molecules.

## Summary

- Sulfation of small molecules is implicated in critical biological signalling cascades and a key phase II drug metabolism step.
- Methods to prepare sulfated molecules to enable biological study are both limited and have practical disadvantages to isolate tractable quantities of the desired sulfate.
- Emerging methods that build upon the early work of amine-sulfur trioxide complexes (e.g. tributylsulfoammonium betaine, TBSAB), *in situ* preparation of reactive sulfating complexes, and SuFEx methodology are gaining traction in the sulfation community.

## Abbreviations

DMA: dimethylacetamide
DMS: dimethyl sulfate
DPS: diisopropyl sulfate
NaH: sodium hydride
NaHMDS: sodium hexamethyldisilazide
PPS: pentosan polysulfate
TBSAB: tributylsulfoammonium betaine
TMSC: trimethylsilyl cellulose
